# Dosimetric evaluation of MRI‐guided multi‐leaf collimator tracking and trailing for lung stereotactic body radiation therapy

**DOI:** 10.1002/mp.14772

**Published:** 2021-03-12

**Authors:** Prescilla Uijtewaal, Pim T.S. Borman, Peter L. Woodhead, Sara L. Hackett, Bas W. Raaymakers, Martin F. Fast

**Affiliations:** ^1^ Department of Radiotherapy University Medical Center Utrecht Heidelberglaan 100 Utrecht 3584 CX The Netherlands

**Keywords:** lung cancer, MLC‐tracking, MRI‐linac, prediction, trailing

## Abstract

**Purpose:**

The treatment margins for lung stereotactic body radiotherapy (SBRT) are often large to cover the tumor excursions resulting from respiration, such that underdosage of the tumor can be avoided. Magnetic resonance imaging (MRI)‐guided multi‐leaf collimator (MLC) tracking can potentially reduce the influence of respiration to allow for smaller treatment margins. However, tracking is accompanied by system latency that may induce residual tracking errors. Alternatively, a simpler mid‐position delivery combined with trailing can be used. Trailing reduces influences of respiration by compensating for baseline motion, to potentially improve target coverage. In this study, we aim to show the feasibility of MRI‐guided tracking and trailing to reduce influences of respiration during lung SBRT.

**Methods:**

We implemented MRI‐guided tracking on the MR‐linac using an Elekta research tracking interface to track tumor motion during intensity modulated radiotherapy (IMRT). A Quasar ${\text{MRI}}^{4D}$ phantom was used to generate Lujan motion (${\text{cos}}^{4}$, 4 s period, 20 mm peak‐to‐peak amplitude) with and without 1.0 mm/min cranial drift. Phantom tumor positions were estimated from sagittal 2D cine‐MRI (4 or 8 Hz) using cross‐correlation‐based template matching. To compensate the anticipated system latency, a linear ridge regression predictor was optimized for online MRI by comparing two predictor training approaches: training on multiple traces and training on a single trace. We created 15‐beam clinical‐grade lung SBRT plans for central targets (8 × 7.5 Gy) and peripheral targets (3 × 18 Gy) with different PTV margins for mid‐position based motion management (3–5 mm) and for MLC tracking (3 mm). We used a film insert with a 3 cm spherical target to measure the spatial distribution and quantity of the delivered dose. A 1%/1 mm local gamma‐analysis quantified dose differences between motion management strategies and reference cases. Additionally, a dose area histogram (DAH) revealed the target coverage relative to the reference scenario.

**Results:**

The prediction filter gain was on average 25% when trained on multiple traces and 44% when trained on a single trace. The filter reduced system latency from 313 ± 2 ms to 0 ± 5 ms for 4 Hz imaging and from 215 ± 3 ms to 3 ± 3 ms for 8 Hz. The local gamma analysis for the central delivery showed that tracking improved the gamma pass‐rate from 23% to 96% for periodic motion and from 14% to 93% when baseline drift was applied. For the peripheral delivery during periodic motion, delivery pass‐rates improved from 22% to 93%. Comparing mid‐position delivery to trailing for periodic+drift motion increased the local gamma pass rate from 15% to 98% for a central delivery and from 8% to 98% for a peripheral delivery. Furthermore, the DAHs revealed a relative $D_{98\%}$ GTV coverage of 101% and 97% compared to the reference scenario for, respectively, central and peripheral tracking of periodic+drift motion. For trailing, a relative $D_{98\%}$ of 99% for central and 98% for peripheral trailing was found.

**Conclusions:**

We provided a first experimental demonstration of the technical feasibility of MRI‐guided MLC tracking and trailing for central and peripheral lung SBRT. Tracking maximizes the sparing of healthy tissue, while trailing is highly effective in mitigating baseline motion.

## 1 INTRODUCTION

Stereotactic body radiation therapy (SBRT) has become a viable alternative for surgery to treat early stage non‐small‐cell lung cancer (NSCLC).^1^, ^2^, ^3^, ^4^ Treatments are characterized by high, ablative doses of radiation in which relatively small target volumes are irradiated in only a few fractions.^1^, ^5^, ^6^ Small target volumes allow for a higher, more effective radiation dose,^7^ while sparing surrounding healthy tissue from radiation‐induced damage.^1^, ^3^, ^6^

A challenge in SBRT for lung tumors is the relatively large respiratory motion, that typically ranges between 1 and 3 cm.^8^, ^9^, ^10^ The classic approach to minimize underdosage of the gross tumor volume (GTV) as a result of this intrafractional motion, is to build an internal target volume (ITV) that covers the full tumor motion excursions.^11^ However, the resulting planning target volume (PTV) is large and might overlap with mediastinal structures near central tumors. High‐dose radiation treatments of central tumors may lead to severe toxicities in these mediastinal structures,^4^, ^12^ meaning only a lower, less effective dose can be used to treat central tumors.^7^ Even though peripheral tumors are far away from critical organs, it is still desirable to spare the lung itself as much as possible. To increase the applicability of SBRT for lung tumors, treatments require utmost precision with smaller treatment margins.^9^, ^13^, ^14^

To increase the treatment precision, the delivery of lung SBRT should be adapted in real‐time according to the observed respiratory motion. This requires continuous information about the patient’s anatomy.^2^ The Unity MR‐linac (Elekta AB, Stockholm, Sweden) features a 1.5 T magnetic resonance imaging (MRI) scanner to monitor tumor excursions in real‐time with high contrast images. Additionally, it is equipped with a multi‐leaf collimator (MLC) that enables real‐time treatment adaptations by moving its leaves.^15^

The real‐time monitored tumor positions can facilitate tumor tracking, in which the monitored tumor positions are used to continuously realign the treatment beam with the tumor position using the MLC.^9^, ^14^ Previous experiments demonstrated MRI‐guided MLC tracking on the Unity MR‐linac for a single gantry angle with a conformal treatment field as a proof‐of‐principle.^16^ An *in silico* study proved the feasibility of MLC tracking for clinically acceptable lung SBRT.^9^ These experiments revealed that tracking increases the dose delivery accuracy, showing the potential to use smaller treatment margins.^9^ However, this has not yet been demonstrated in a phantom dosimetric experiment. Performing MRI‐guided MLC tracking in a phantom dosimetric experiment is challenging because there is a time lag between the physical motion and the MLC response: the system latency.^14^, ^16^, ^17^ System latency is problematic during tracking because it creates inaccuracies, causing the beam to miss the GTV and to irradiate healthy tissue instead.^14^, ^16^ Although MLC tracking consists of several steps that all induce latency, the main contributor to the system latency is the MRI acquisition process.^16^ The relatively low imaging frequency of MRI substantially contributes to the system latency.^14^ Previous studies proposed a prediction filter to mitigate the latency during tracking on conventional treatment platforms.^18^, ^19^, ^20^

A less complex alternative to MLC tracking is a mid‐position (midP) delivery in combination with trailing. In a midP delivery, the PTV‐volume corresponds to the time‐weighted average position of a target volume during a full breathing cycle.^11^ This means that the GTV‐to‐PTV margin that is applied is larger than for MLC tracking, but smaller than the conventional margins. Trailing is a technique in which the beam aperture is continuously adjusted according to the target’s last available time‐averaged position.^21^ The benefit of this approach is the simplicity; it only requires a low imaging frequency, and it is insensitive to latency. The continuous adjustment of the beam aperture limits the influence of baseline drift^21^ and therefore potentially improves target coverage compared to a conventional midP delivery.

This study investigates the feasibility of two different motion management strategies to mitigate the influence of respiratory motion during MRI‐guided lung SBRT: MLC tracking, and a midP delivery in combination with trailing. We carried out three lines of experiments to investigate this question. First, we created a prediction filter optimized for low frequency, online MRI‐guidance to mitigate system latency. *In silico* experiments were used to identify the best predictor training strategy with the highest prediction accuracy. Second, latency experiments were carried out to determine if our prediction filter can compensate for latency during image‐guided MLC tracking. Third, intensity‐modulated radiotherapy (IMRT) plans were generated for tracking and for a midP delivery using the clinical template for lung SBRT. These plans were applied in phantom experiments with film dosimetry to dosimetrically evaluate the performance of both motion management strategies for central and peripheral tumors during lung SBRT.

## 2 MATERIALS AND METHODS

### 2.1 Experimental setup

All tracking and trailing experiments were performed on a Unity MR‐linac (Elekta AB, Stockholm, Sweden), featuring a 7 MV Linac and a 1.5 T MR scanner with its main magnetic field oriented perpendicularly to the beam direction. A 160‐leaf MLC, with fixed collimator angle at $270^{\circ }$, was used to dynamically shape the radiation beam in the superior‐inferior (SI) direction, while additionally confining the radiation orthogonal to the beam direction with diaphragms.

**Fig. 1:**
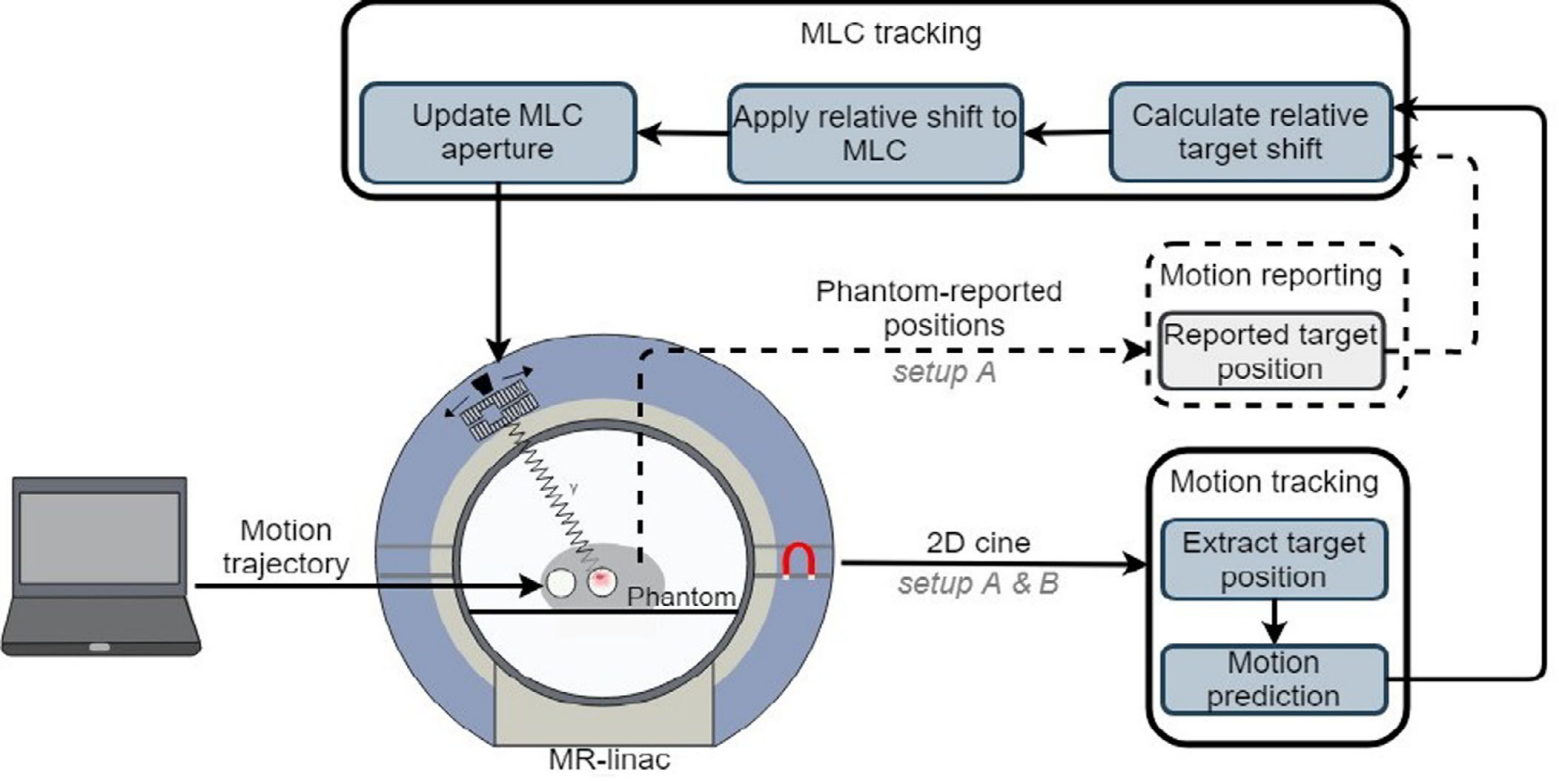
Experimental setup for magnetic resonance imaging‐guided multi‐leaf collimator tracking. Setup A represents the latency setup and is indicated by both the solid and dashed arrows. Setup B represents the dosimetrysetup and is indicated by the solid arrows.

Two different experimental setups were used to perform latency and dosimetry experiments. Both setups contain a Quasar ${\text{MRI}}^{4D}$ phantom (Modus Medical Devices Inc., London ON) in the scanner bore. The experimental setups are depicted in Fig. 1.

#### 2.1.1 Tracking

During MRI‐guided MLC tracking, the leaf velocity and positions were updated in real time with a 40 or 80 ms control system cycle (CSC), using a feed‐forward mode of $K_{\mathrm{FF}}=0.5$ for the conventional proportional‐integral‐differential (PID) leaf motor control.^16^

The continuously updated MLC apertures were transferred to the MLC via the vendor‐provided Elekta digital Linac interface (EDLI). The in‐house developed tracking control software was deployed on a workstation computer equipped with an eight‐thread Xeon E3‐1240, 16 GB of memory and running Debian 9. The tracking software contains two threads that run asynchronously with each other. One thread receives the MR images from the scanner and calculates the target position (4 or 8 Hz), while the other thread takes the most recent position and sends the corresponding MLC aperture update to the machine (12.5 or 25 Hz). This asynchrony induces a varying system latency.

To mitigate system latency during MLC tracking, the aperture was shifted according to predicted positions using a prediction filter, as will be described in Section 2.B.

#### 2.1.2 Trailing

For trailing, we used the exact same setup as for tracking, but with a different position input signal. Here the MLC aperture was continuously shifted according to the target’s last available time‐averaged position averaged over three respiratory cycles.^21^ For trailing, MLC apertures were updated using a 80 ms CSC.

#### 2.1.3 Latency setup

The main contributor to geometrical errors in image‐guided MLC tracking of a respiratory motion target is the system latency,^22^ which can be defined as the difference between the time of a physical motion event and the time to change the MLC aperture position in reaction to this event. The first experimental setup (A) was used to measure this system latency. In the setup, the phantom contained a cylindrical acrylic container filled with ${\text{MnCl}}_{2}$‐doped agar gel (3% solution). A circular ${\text{ZrO}}_{2}$ ball bearing (10 mm diameter) was fixated in the center of the plastic container by a stem that was extended by a three‐dimensional (3D) printed bar clamping the ball bearing. A ceramic ball bearing was chosen because it provides contrast in both MRI and X‐ray based imaging, while it avoids MRI artefacts. The phantom was programmed with sinusoidal motion with a 20 mm peak‐to‐peak amplitude and a 4 s period in the superior‐inferior (SI) direction. The current position was reported and streamed to the tracking software by the phantom with negligible latency (<1 ms). In this experimental setup, a circular aperture of 5 cm diameter that could be translated in the SI direction was applied.

Based on theoretical grounds, the minimum latency ($\tau_{\min}$) depends on both the signal acquisition time ($T_{\mathrm{signal}}$), that is, the elapsed time from acquiring the center of k‐space ($k_{0}$)^23^ until the image is received, the image processing time ($T_{\mathrm{proc}}$), that is, the necessary time to both extract the target position from the image to calculate the relative target shift and to calculate the corresponding MLC aperture, and the actual MLC adjustment time ($T_{\mathrm{MLC}}$).^17^, ^24^ This can be expressed as^14^

(1) $$\tau_{\min}=T_{\mathrm{signal}}+T_{\mathrm{proc}}+T_{\mathrm{MLC}}.$$

$T_{\mathrm{signal}}$ was measured by extracting the time‐shift between the phantom‐reported positions and the MR image‐derived positions from the log files. The value for $T_{\mathrm{proc}}$ can be derived from the MRI log files.

The (average) system latency and $T_{\mathrm{MLC}}$ were estimated using an integrated electronic portal imaging device (EPID) panel (C = 0.25 pF, 33 ms integration time, X/2‐binning). The EPID images show the positions of both the high‐contrast ${\text{ZrO}}_{2}$ target and the tracked circular MLC‐aperture during the sinusoidal motion performed by the motion stage. The positions of both quantities were deduced from every frame of this image series.^17^ Accordingly, a sinusoidal model was fit to both quantities:

(2) $$P_{\mathrm{MLC}}\left(t\right)=Asin\left(2\pi t+\phi_{0}\right),$$

(3) $$P_{\mathrm{target}}\left(t\right)=Asin\left(2\pi \left(t‐\Delta T\right)+\phi_{0}\right),$$

where $P_{\mathrm{MLC}}$ denotes the MLC‐aperture positions, $P_{\mathrm{target}}$ denotes the target positions, *A* denotes the amplitude, and *ϕ* denotes the phase. Based on an EPID series of around 30 s, the time shift between $P_{\mathrm{MLC}}$ and $P_{\mathrm{target}}$ was used to estimate the system latency ΔT. The system latency was determined for both 4 and 8 Hz imaging, as well as for a 40 and 80 ms CSC. The value for $T_{\mathrm{MLC}}$ was measured by estimating the system latency when phantom‐reported positions were fed to the MLC‐tracking software ($T_{\mathrm{signal}}$ = 0), instead of image‐derived positions.

#### 2.1.4 Dosimetry setup

A second setup (B) was used for film dosimetry measurements to evaluate the performance of both MLC‐guided tracking and trailing. Here the cylindrical acrylic insert of the phantom was replaced by a prototype film dosimetry insert. This insert consisted of a similar acrylic container filled with ${\text{MnCl}}_{2}$‐doped agar gel, and of a film cassette that could be fitted into the cylinder. A 3 cm diameter spherical target was located just above the cassette opening, in the center of the container. The body oval, in which the cylinder was placed, was filled with ${\text{MnCl}}_{2}$‐doped water. During the experiments, the motion stage performed no motion, ${\text{cos}}^{4}$ Lujan motion with 20 mm peak‐to‐peak amplitude and 4 s period, or Lujan motion with additional 1.0 mm/min baseline drift in SI direction. The aperture shape was read from the treatment plan.

#### 2.1.5 MR imaging

For both experimental setups, the phantom positions were continuously estimated from sagittal 2D cine‐MR that was acquired using a T1‐weighted gradient echo (GRE) sequence. The sequence parameters were chosen as follows for 4 Hz (8 Hz) imaging: $350\;\times \;350\;{\text{mm}}^{2}\;(400\;\times \;207\;{\text{mm}}^{2}$) field of view (FOV), $2.5\;\times \;2.5\;{\text{mm}}^{2}\;(3\;\times \;3\;{\text{mm}}^{2}$) voxel size, 10 mm (15 mm) slice thickness, echo time ($T_{E})\;=\;2\;ms\;(2.7\;ms)$, repetition time ($T_{R})\;=\;4\;ms\;(1.44\;ms)$, flip‐angle = $6^{\circ }$, acquisition time ($T_{\mathrm{acq}})\;=\;252\;\text{ms}\;(129\;\text{ms})$. The reconstructed images were continuously streamed, in real‐time, to the tracking software using the vendor‐provided MRTC interface. This external control interface enables the MR‐linac’s therapy control software to acquire MR images as part of the MRI‐guided treatment workflow. The target position was estimated using a cross‐correlation based template matching algorithm.^25^, ^26^, ^27^

### 2.2 Prediction Model

Forward prediction of breathing motion can compensate for the system latency and it improves the accuracy of image‐guided MLC tracking.^22^ However, the system latency can vary, because the image receiver thread and the MLC tracking thread run asynchronously, and because the imaging receiver thread runs at a lower frequency than the MLC tracking thread. Whereas previous studies compensated the average system latency,^14^, ^16^ we aim to compensate the current system latency, calculated based on $k_{0}$ of the last received image. This was done using a linear (ridge) regression model that can be trained in real‐time to predict future tumor positions. For every new tumor position, the prediction filter predicted position 250 and 500 ms (250 and 375 ms for an 8 Hz predictor) ahead, such that we know three tumor states: last reported position, first predicted position, and second predicted position. Depending on the current system latency, the desired tumor position can be continuously interpolated between the two suitable states. A linear (ridge) regression model was chosen since it is a relatively simple and fast model that predicts respiratory motion with similar accuracy as more complex models.^19^

#### 2.2.1 Respiratory data

For the development and optimization of the prediction model, 94 breathing data samples from the study of Sun et al (2008)^28^ were used. In their study, the respiratory trajectories of 30 patients were estimated by a Cyberknife Synchrony system (Accuray Inc., Sunnyvale, CA). In our study, only motion in SI direction was considered.

Each data sample was expressed as uniformly sampled time series, linearly interpolated on either a 4 Hz or 8 Hz time grid $\left\{s_{i}:={\left(s\left(t_{i}\right)\right)}^{T}|i\in \left[1,N\right]\right\}$, such that for each discrete time point $t_{i}$, a tumor position $s_{i}$ was derived, for the total number of recorded positions (N).

#### 2.2.2 Data preprocessing

Before the prediction, data preprocessing was performed to reduce the influence of baseline drift and large amplitude fluctuations on the prediction accuracy. Based on a growing training window, initially consisting of the last 30 s of tumor positions $\left\{s\left(t_{i}\right)|t_{i}\in \left[t_{i‐N_{\mathrm{train}}},t_{i}\right]\right\}$, the respiratory period was calculated. $N_{\mathrm{train}}$ defined the number of tumor positions in the training window. With every new available position, the growing window grew up till it reached its optimum, after which the window became a sliding window. The respiratory period was calculated by performing a Fourier transformation on the training window, where the non‐zero frequency component $f_{\max}$ is taken as the respiratory period.

The respiratory period was used to define a sliding preprocessing window, that contained the tumor positions of the last two periods $\left\{s\left(t_{i}\right)|t_{i}\in \left[t_{i‐N_{\mathrm{sw}}},t_{i}\right]\right\}$. $N_{\mathrm{sw}}$ denotes the number of positions within the sliding preprocessing window. This window was normalized to have zero mean and unity variance.^19^

The normalized tumor positions $s_{i,\mathrm{norm}}$ were used to construct pairs of input vectors and target scalars $\left\{\left(x_{i},y_{i}\right)|\in \left[1,N\right]\right\}$, whereby the subscript *i* defines points in time $t_{i}$. From the history of past tumor positions $\left\{s_{i}|i\leq j\right\}$, the input vectors $x_{i}$ were constructed. The target scalar $y_{i}$ depended on the lookahead length *τ*, and was defined as the tumor position *τ* time steps ahead: $y_{j}=s_{j+\tau }$. Because we calculate two predictions for every input vector $x_{i}$ each having a different lookahead length, two separate scalars were defined. The lookahead lengths were chosen such that *τ* was always an integer multiple of the sampling interval.

#### 2.2.3 Linear (ridge) regression

For the final prediction, a linear regression function $f\left(x\right)=\beta^{T}x$ with weight vector *β* was used to describe a multi‐dimensional linear map of the input vector, to obtain an estimate of the future target position. The weight vector *β* needs to be trained with previously described input vectors and target scalars using ridge regression.^19^ It is important to note that the prediction is based on normalized data, resulting in a normalized predicted position ${\hat{y}}_{\mathrm{norm}}$. Therefore, ${\hat{y}}_{\mathrm{norm}}$ needed back‐transformed using the stored normalization values to obtain the actual predicted position $\hat{y}$.^19^

#### 2.2.4 Predictor training strategies

To investigate the trade‐off between prediction robustness and specificity, we investigated two different approaches to training the linear regression predictor. In the first approach, the predictor trained offline on multiple training trajectories, each providing a weight vector. From these weight vectors, an average weight vector was calculated, such that it became more robust against fluctuations in amplitude and period. In this study, a leave‐one‐out cross‐validation method was used, whereby the predictor trained on 93 of the 94 respiratory traces. The last independent respiratory trace was not used for training, but was instead used to test the prediction. In this approach, the predictor is only trained once on a large amount of respiratory traces.

In the second approach, only a single respiratory trace was used for both online training and predicting. To do so, the previously described training window, initially consisting of the last seconds of tumor positions, was not only used to calculate the respiratory period, but also to train the predictor. In this approach, the predictor is retrained every time a new target position becomes available. Depending on the sampling frequency (4 or 8 Hz), this can be every 125 or every 250 ms. The second approach allowed for two types of retraining: training on a sliding training window or training on a growing training window. Both approaches started with a stationary window, consisting of the last seconds of target positions, such that the predictor could always train on a sufficient amount of data.

The prediction accuracy was quantified in terms of a root‐mean‐squared error (RMSE) between the actual target position $y_{j}=s_{j+\tau }$ and the back‐transformed predicted position $\hat{y_{j}}$.

#### 2.2.5 Statistics

The Spearman correlation test was used to identify the influence of individual respiratory characteristics, for example, amplitude and period, on the prediction accuracy.

A one‐way ANOVA was used to compare the prediction performance: without predictor, with predictor using multiple respiratory traces for training, and with predictor using a single respiratory trace for training. Tukey’s test was used for post hoc comparisons. A two‐sided Wilcoxon signed‐rank test was performed to compare the prediction performance in terms of RMSE of a growing window to a sliding window. For both tests, a significance level of 0.05 was used.

### 2.3 Dosimetric accuracy

For the assessment of the dosimetric benefits of both MRI‐guided trailing and MRI‐guided MLC tracking, ${\text{Gafchromic}}^{\mathrm{TM}}$ radiochromic films placed in the phantom’s film cassette (setup B), were irradiated. EBT3 films were used for the central delivery plans and EBT‐XD films were used for the peripheral delivery plans, because these films can cover a larger dose range.

#### 2.3.1 Treatment planning

A 15‐beam tracking and a 15‐beam midP treatment plan was created for both the central and the peripheral cylinder location using the clinical template for lung SBRT using Monaco 5.40 (Elekta AB, Stockholm, Sweden) as treatment planning system. The plans were created by an experienced clinical physicist. For delineation, a planning CT of the phantom with a 1$\;\times \;1\;\times \;1.5\;{\text{mm}}^{3}$ voxel size was acquired on the Brilliance Big Bore CT scanner (Philips Medical Systems, Best, The Netherlands) for both cylinder locations. The 3 cm diameter spherical target was delineated as GTV. The PTV was obtained by adding an isotropic margin of 3 mm in all directions for the tracking delivery, and for the midP delivery anisotropic margins of 3 mm left‐right, 4 mm anterior‐posterior, 5 mm SI direction were used based on the anticipated respiratory motion. A dose of 7.5 Gy in 8 fractions, and a dose of 18 Gy in 3 fractions was prescribed for the PTV for, respectively, the central and the peripheral delivery. The number of segments was constrained to 40 (center cylinder location) or 45 (peripheral cylinder location) to ensure that segments for tracking plans were at least two leaves wide. Plans were calculated with a 3 mm grid size and 3% Monte Carlo uncertainty per control point.

#### 2.3.2 Treatment delivery

Three motion scenarios were investigated: static (no phantom movement), periodic phantom motion, and periodic + baseline phantom motion. Periodic motion was defined as ${\text{cos}}^{4}$ Lujan motion, with a 20 mm peak‐to‐peak amplitude and 4 s period. The applied baseline drift was a 1.0 mm/min continuous linear drift in SI direction. For tracking, we compared a static delivery to a delivery: without tracking, tracking without prediction filter, and tracking with prediction filter. To investigate the effect of trailing, we compared a conventional midP delivery for periodic motion to both a conventional midP delivery and to a midP+trailing delivery for periodic motion with additional baseline drift.

#### 2.3.3 Dosimetric evaluation

The irradiated films were scanned and digitized with an Epson Expression 10000XL flatbed scanner (Seiko Epson Corp, Nagano, Japan). The digitized films were analyzed using inhouse‐developed software. The films were registered based on three indents in the corner of each film that were created by the phantom’s film cassette. A semi‐automatic indent detection feature localized these indents and registered the film using a point matching algorithm. The correspondence between the dose distributions of the registered films were analyzed using a local gamma‐analysis with three separate gamma evaluation criteria: 1% dose/1 mm distance to agreement (DTA), 2% dose/2 mm DTA, and 3% dose/3 mm DTA.^29^ Only pixels with >10% prescribed dose were included in the analysis, such that pixels with large film calibration uncertainties were excluded.

The GTV‐coverage was quantified using dose area histograms (DAHs), whereby at each dose *d*, the percent area of the GTV that is exposed to ≥ *d* is defined as *v(d)*.

## 3 RESULTS

### 3.1 Predictor training

#### 3.1.1 Training approaches

**Fig. 2:**
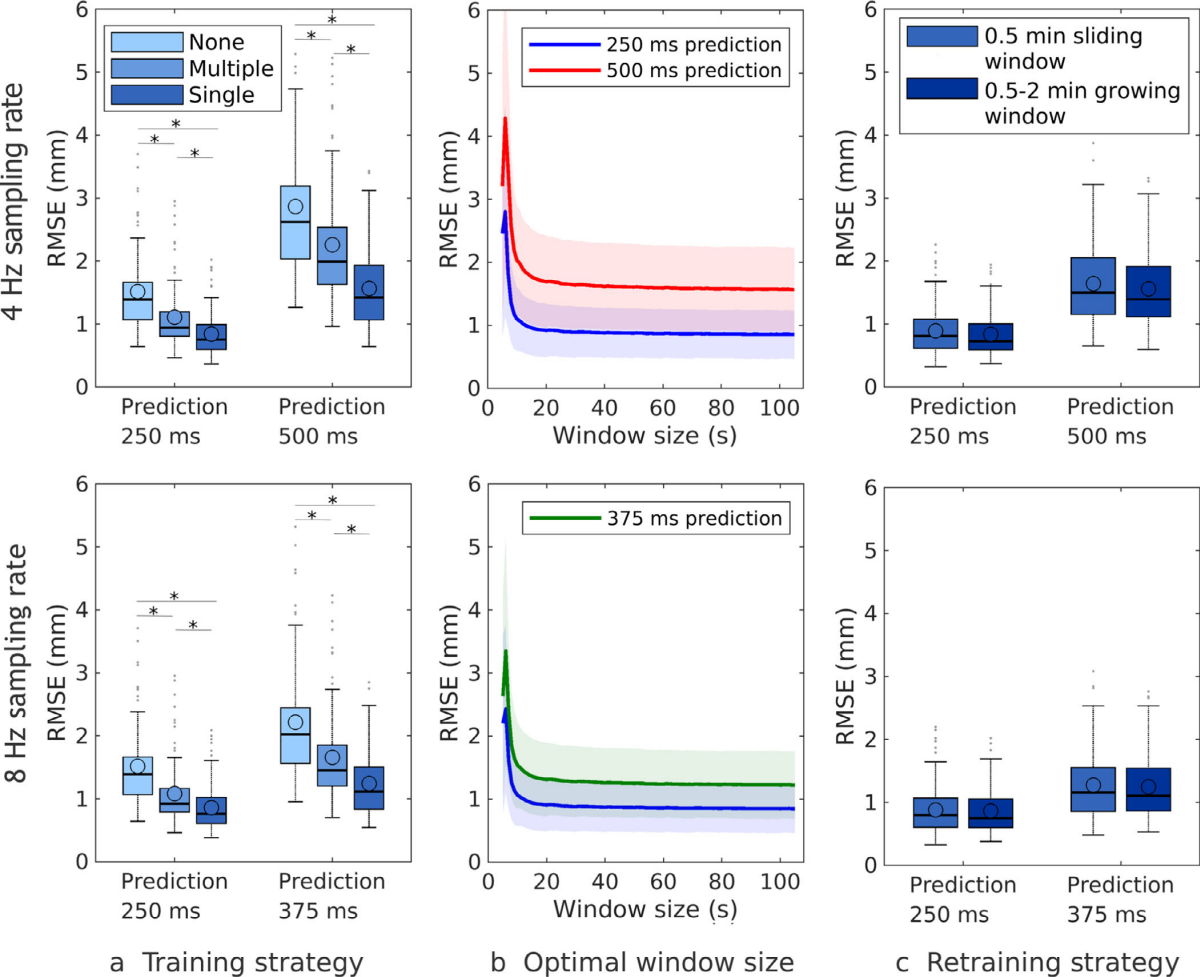
Comparison of prediction accuracy between different training approaches for a 4 and 8 Hz sampling rate. (a, b) Prediction performance of different training strategies. (c) Prediction performance for different amounts of training data.

The geometric accuracy gain of the predictor using different types of training is shown in Fig 2(a). The prediction method “none" indicates that the last position observation was used as prediction value. The use of a predictor reduced the RMSE (*P* < 0.05) on average between 25% and 44% for predictions with a 250 ms lookahead length compared to the no‐prediction scenario. Furthermore, it can be seen that prediction training on a single respiratory trace gave more accurate results (*P* < 0.05) than training on multiple traces. Because training on a single respiratory trace resulted in a better prediction performance, we further analyzed the effect of training using this approach.

Figure 2(b) shows the prediction performance in terms of RMSE for different training window sizes. Initially, the prediction performance improved, when the size of the training window increased. Once the training window contained 30 s of data, the increase in prediction accuracy became neglectable. Because of this observation, further analysis was done using a 30 s training window.

Figure 2(c) shows the performance of a predictor that was trained on either a 30 s sliding window or growing window. It can be seen that training on a growing window, containing more data over time than the sliding window, gave a marginally better prediction performance than training on a sliding window. Using this training approach in the experimental setup for film dosimetry for a 4 Hz imaging frequency, we obtained an RMSE of 0.1 and 0.2 mm for lookahead lengths of, respectively, 250 and 500 ms for the Lujan trajectory.

#### 3.1.2 Influences on prediction performance

**Fig. 3:**
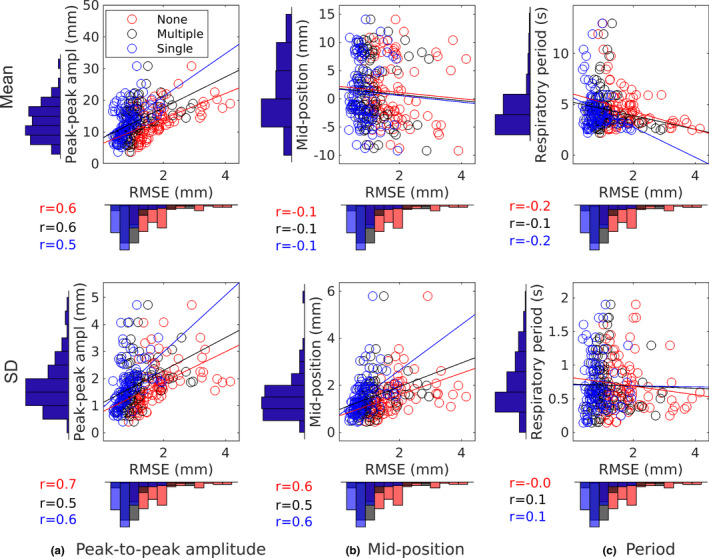
Influence of respiratory characteristics on prediction performance of different predictor training approaches for a 250 ms lookahead length and a 4 Hz sampling rate. Histograms show the distribution of the respiratory characteristics and of the root‐mean‐squared error scores. Respiratory characteristics are presented in terms of mean (top row) and SD (bottom row).

Figure 3 shows the correlation between respiratory characteristics and prediction performance for 4 Hz sampled data, a growing training window, and predictions with a 250 ms lookahead time. Displayed are three prediction approaches: no prediction, prediction training on multiple respiratory trajectories, and training on a single trajectory. As shown the respiratory amplitude moderately influenced prediction performance whereby larger peak‐to‐peak amplitudes, as well as amplitude deviations resulted in slightly higher RMSE values. Figure 3(b) shows that the midP (average amplitude height) on average did not influence prediction performance. However, some moderate influence could be observed for deviations from the midP. Figure 3(c) shows that respiratory period did not influence prediction performance. Similar correlations were found for predictions with a lookahead length of 500 ms as well as for 8 Hz sampled data.

### 3.2 System latency

The MRI log files revealed a $\tau_{\mathrm{proc}}$ of 10 ms. Table I summarizes the results of the individual latency components, as well as the measured latency values for measurements with and without a prediction filter. Going from 4 to 8 Hz cine‐MRI reduces $T_{\mathrm{signal}}$ by 45 ms and $\tau_{\mathrm{MLC}}$ by 8 ms. Increasing the imaging frequency reduces the MLC tracking system latency by approximately 100 ms, while the impact of CSC on the measured latency was minimal. Note that the system latency was not fully compensated by the predictor for 4 Hz imaging with a 40 ms CSC. The remaining system latency and the small impact of CSC is a result of queuing, which we observed for the 40 ms CSC. Queuing occurs when EDLI receives new apertures faster than it can process them, leaving a queue with new apertures that start to age. For the other scenarios the predictor effectively mitigated the system latency. Because an 80 ms CSC resulted in the lowest and most stable system latency for 4 Hz imaging, this setting was used in the dosimetry experiments.

**Table I:** System latency with and without prediction filter. The SD is reported between brackets

$f_{\mathrm{MRI}}$ (Hz)	CSC (ms)	$T_{\mathrm{signal}}$ (ms)	$\tau_{\mathrm{MLC}}$ (ms)	$\tau_{\min}$ (ms)	$\tau_{\mathrm{measured}}$ (ms)	$\tau_{\mathrm{measured}}^{w/predictor}$ (ms)
4	40	103 (±1)	92 (±2)	205 (±2)	323 (±2)	12 (±1)
4	80	103 (±1)	100 (±5)	213 (±5)	313 (±2)	0 (±5)
8	40	58 (±1)	92 (±2)	160 (±2)	213 (±2)	0 (±2)
8	80	58 (±1)	100 (±5)	168 (±5)	215 (±3)	3 (±3)

### 3.3 Dosimetry analysis

Treatment delivery times were 6.2 and 9.9 min for the central and peripheral lung SBRT plans, resulting in a total baseline drift of 6.2 mm and 9.9 mm for the periodic+drift motion scenario.

For the tracking plans, an average registration error of 0.5 ± 0.3 mm was found for the central plans, and of 0.3 ± 0.3 mm for the peripheral plans. The registration of the midP plans was accompanied by an average error of 0.6 ± 0.3 and 0.6 ± 0.4 mm.

#### 3.3.1 Dose profiles

**Fig. 4:**
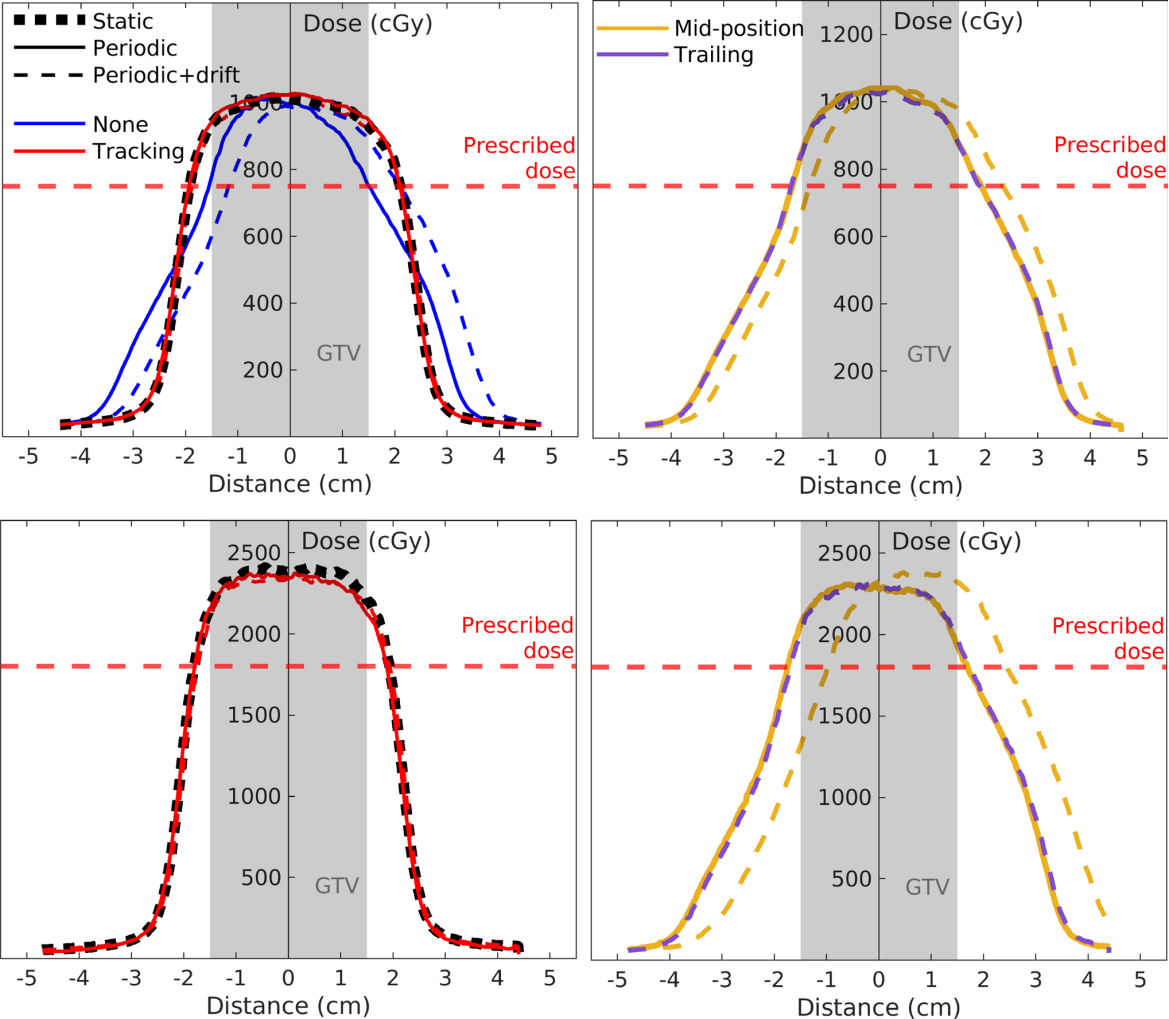
Dose profiles for film measurements of different motion management strategies. The line type indicates the motion scenario and the line color represents the used motion management strategy. Tracking scenarios were compared to a static delivery as a reference(black dashed line), and mid‐position deliveries w/ and w/o trailing during periodic+driftmotion were compared to a periodic delivery as a reference (yellow solid line). None refers to a scenario where the tracking plan was delivered, but no actual tracking was applied. Note that the no tracking scenario is only shown for the central delivery. Also note that the red lines representing the tracking scenario are superimposed.

Dose profile comparisons in SI direction between film measurements of different motion scenarios are shown in Fig. 4. In the tracking scenario, the static line represents the reference case. The dose profiles of the central delivery reveal a loss of GTV coverage when no tracking was applied for periodic motion with and without baseline drift. Furthermore, they show that the dose profile without tracking deviates substantially from the static reference. Applying tracking vastly improved the GTV coverage. For both motion scenarios, we also see that the dose profile of tracking accurately follows the reference profile.

For the MidP delivery, the periodic motion case is used as a reference. The dose profiles show that additional baseline drift shifted the dose profile with the time‐averaged applied drift, decreasing the GTV coverage. Trailing compensated for this additional baseline drift, giving a dose profile similar to the periodic midP scenario.

#### 3.3.2 Gamma analysis

**Fig. 5:**
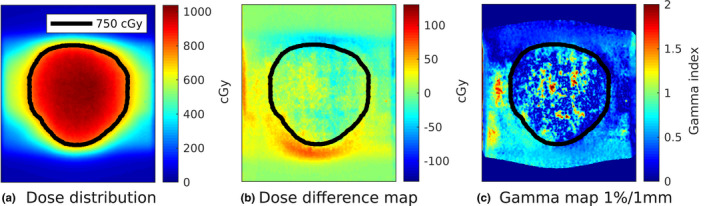
Dosimetric maps of central tracking delivery with predictor for periodic motion with additional baseline drift. The black line represents the prescription iso‐dose line.

Figure 5 shows an example of the dosimetric maps for a central tracking scenario with periodic motion and baseline drift. For both motion scenarios and for all motion management approaches, the percentages of pixels that passed or met the local gamma criteria: 1%/1, 2%/2, and 3%/3 mm are provided in Table II. It can be seen that tracking, with and without prediction filter, increased the gamma passing rate, whereby highest values were obtained when a prediction filter was used. For all scenarios, tracking shows higher gamma passing rates compared to midP delivery. For periodic+drift motion, the local gamma passing rate increased using trailing compared to a midP deliver for all gamma criteria. However, Fig. 5(b) shows that even for the perfect tracking case we have some small residual differences compared to the static reference.

**Table II:** Gamma passing rates for pixels >10% prescribed dose comparing motion management strategies for periodic motion with and without added baseline drift to a static reference case. None refers to a scenario where the tracking plan was delivered, but no actual tracking was applied.

	Periodic motion			Periodic+drift motion		
	1%/1mm	2%/2mm	3%/3mm	1%/1mm	2%/2mm	3%/3mm
Central delivery						
Tracking						
none	23	44	58	14	29	44
w/o predictor	59	83	97	–	–	–
w/ predictor	96	100	100	93	100	100
Mid‐position						
conventional	–	–	–	15	29	48
trailing	–	–	–	98	99	100
Peripheral delivery						
Tracking						
none	22	44	58	–	–	–
w/o predictor	40	75	97	–	–	–
w/ predictor	93	100	100	88	99	100
Mid‐position						
conventional	–	–	–	8	15	22
trailing	–	–	–	98	100	100

#### 3.3.3 Dose area histograms

Table III summarizes the dosimetric outcomes for all treatment deliveries and motion scenarios. The main differences in target coverage were obtained for the minimum dose ($D_{98\%}$), while the median dose ($D_{50\%}$) and the maximum dose ($D_{2\%}$) were similar for all scenarios. For all static deliveries, the GTV coverage was well above the prescribed dose, as was expected. These GTV coverages were preserved during periodic motion w/ or w/o additional baseline drift when tracking with a prediction filter was applied. When no tracking was applied, the $D_{98\%}$ target coverage decreased by 20% and 23% for respectively a central and peripheral target during periodic motion. During periodic motion w/ additional baseline drift, the decrease in target coverage for a central target was 27%.

**Table III:** Dose area histogram comparison between motion management strategies for periodic motion with and without additional baseline drift relative to a static reference case. None refers to a scenario where the tracking plan was delivered, but no actual tracking was applied.

	DAH GTV			Periodic motion			Periodic+drift motion		
	$D_{98\%}$ (Gy)	$D_{50\%}$ (Gy)	$D_{2\%}$ (Gy)	$D_{98\%}^{\mathrm{rel}}$ (%)	$D_{50\%}^{\mathrm{rel}}$ (%)	$D_{2\%}^{\mathrm{rel}}$ (%)	$D_{98\%}^{\mathrm{rel}}$ (%)	$D_{50\%}^{\mathrm{rel}}$ (%)	$D_{2\%}^{\mathrm{rel}}$ (%)
Central delivery									
Tracking									
static	9.0	9.8	10.2	–	–	–	–	–	–
none	–	–	–	80	96	99	72	94	95
w/o predictor	–	–	–	96	98	99	–	–	–
w/ predictor	–	–	–	101	100	101	101	99	101
Mid‐position									
static static	9.0	9.8	10.1	–	–	–	–	–	–
conventional	–	–	–	99	101	104	81	101	104
trailing	–	–	–	–	–	–	99	100	103
Peripheral delivery									
static	20.5	23.0	24.0	–	–	–	–	–	–
None	–	–	–	87	94	99	–	–	–
w/o predictor	–	–	–	94	97	98	–	–	–
w/ predictor	–	–	–	98	99	99	97	98	98
Mid‐position									
static	20.6	22.3	23.1	–	–	–	–	–	–
conventional	–	–	–	96	99	100	68	100	103
Trailing	–	–	–	–	–	–	98	99	100

MidP delivery has a relatively low GTV‐coverage with a $D_{98\%}$ of 68% for motion scenarios with additional baseline drift. Trailing improves this coverage such that a similar dose was delivered for periodic+drift motion as for periodic motion during a midP delivery, showing that trailing can compensate for baseline drift.

## 4 DISCUSSION

Our experiments demonstrated the feasibility of MRI‐guided MLC tracking and trailing for central and peripheral lung SBRT on the MR‐linac for clinically acceptable IMRT plans. First, we developed a linear regression prediction filter specifically optimized for online MRI‐guidance. The results demonstrated that this filter effectively reduces the system latency to net zero and residual targeting RMSE accompanied with tracking. Then, we showed that the prediction filter substantially reduces the dosimetric errors during motion tracking experiments. Additionally, we showed that trailing prevents underdosage of the GTV that can occur as a result of baseline motion during a midP delivery.

MRI‐guided tracking is challenging because of the relatively low imaging frequency (4 or 8 Hz) that causes the MLC to track an outdated tumor position rather than the current position. Aside from the imaging interval, also the signal acquisition time, the image processing time, the MLC adjustment time, and half the CSC interval contribute to the system latency that increases tracking errors.^14^, ^30^ Because the imaging and delivery system run asynchronously, the system latency varies per tracking cycle. A prediction filter compensates for this varying system latency by predicting the tumor position 250 and 500 ms (250 and 375 ms for an 8 Hz predictor) ahead, such that the tracking controller knows three tumor states: last reported position, first predicted position, and second predicted position. This range was used as we anticipated working on the MR‐linac for which latency values remain under 500 ms.^16^ Depending on the current system latency, the desired tumor position is then continuously interpolated between the three tumor states, instead of always compensating the average system latency as was done in previous studies.^14^

Prediction of lung tumor motion during respiration is often challenging because of irregular and complex breathing patterns^3^, ^30^ that might lead to inaccuracies during MLC tracking. Therefore, we evaluated the influence of these irregularities on prediction performance in *in silico* experiments. We found a linear regression predictor to be applicable to a wide range of respiratory periods and amplitudes. However, the larger the amplitude or the amplitude fluctuations, the more challenging the prediction becomes for the predictor. Preprocessing the data before applying a predictor eliminates amplitude variations and baseline shifts that can distort the prediction.^19^ However, large amplitude fluctuations or sudden, instantaneous baseline drifts can only be reduced, meaning that normalized data can still contain some variable amplitudes.

Other than the type of predictor, the type of training also determines the performance of the prediction filter.^19^, ^20^ Online training on a single respiratory trace gave a better performance compared to offline training on multiple respiratory traces. Moreover, for MLC tracking a trace specific predictor is most desirable, because it allows for a very patient specific and adaptive treatment. Training on only a single trace is a new training approach whereby the predictor is continuously retrained on the last 2 min of a respiratory trace, while predictions are performed each newly detected target position. This creates a trace specific predictor with an average RMSE for a 4 Hz sampling rate of 0.8(± 0.4) and 1.6(± 0.6) mm for lookahead lengths of, respectively, 250 and 500 ms. Online retraining was applied to account for sudden transitions in the breathing pattern,^19^, ^31^ which is particularly beneficial for trajectories with large baseline shifts.^30^ In general, prediction performance decreases as the sampling frequency decreases or if the lookahead length increases.^18^, ^20^ In our study, we used relatively low sampling frequencies of 4 and 8 Hz because cine‐MRI acceleration on Unity is currently constrained by the limited number of receiver coils in combination with the desired high in‐plane resolution. Interestingly, despite the low sampling rates, the performance of our predictor was comparable to other linear regression prediction models.^18^, ^19^ This is supported by studies that found linear predictors to be insensitive to the sampling rate.^18^, ^19^, ^20^

We quantified the MLC tracking system latency on the MR‐linac using a dedicated 3D‐printed phantom setup. As expected based on the results reported by Glitzner et al. (2019), we found a higher system latency of 323(± 2) ms for 4 Hz imaging than for 8 Hz imaging, for which we found a system latency of 213(± 2) ms when using a 40 ms CSC. However, they found a 10 ms lower system latency compared to ours for 8 Hz imaging and a 25 ms higher system latency for a 4 Hz imaging frequency. These differences can be explained by the different motion and tracking settings that were used (motion amplitude = 30 mm, period = 5 ms, $K_{\mathrm{FF}}$ = 0 or 1, 4 Hz imaging sequence by signal averaging). For the 8 Hz imaging frequency, the difference in feed‐forward mode ($k_{\mathrm{FF}}$) explains our higher system latency. The feed‐forward mode directly controls the motor velocity per MLC‐leaf,^16^ meaning a higher $k_{\mathrm{FF}}$ would result in lower latency. The lower system latency we found at a 4 Hz imaging frequency can mainly be explained by the difference in imaging sequence. Glitzner et al. (2019) used signal averaging to turn an 8 Hz sequence in a 4 Hz sequence, which increases $T_{\mathrm{signal}}$ by approximately 50 ms.^23^

The prediction filter effectively reduced the system latency for both 4 and 8 Hz imaging. Only for 4 Hz imaging with a 40 ms CSC we obtained small residual latency. This residual latency is most likely due to queuing of requested MLC position. Queuing occurs when EDLI receives new apertures faster than it can process, leaving a queue with new apertures that start to age. This effect induces additional latency to the system. This means that a 40 ms CSC is less stable than an 80 ms CSC during tracking, and could potentially induce more latency than an 80 ms CSC. Therefore, we selected the 80 ms CSC for all dosimetric experiments for a more stable and predictable tracking performance.

To quantify the dosimetric gain of both MRI‐guided trailing and MLC tracking with prediction filter for a clinically acceptable lung SBRT plan, we used a phantom with prototype film dosimetry insert. Applying tracking during periodic motion yields a GTV coverage similar to the reference scenario, while the target is underdosed without tracking with a $D_{98\%}$ target coverage of only 80% for a central target and of 87% for a peripheral target. The median dose ($D_{50\%}$) and the maximum dose $\left(D_{2\%}\right)$ were similar for all tracking scenarios. The dose distribution obtained during tracking with prediction filter was similar to the static reference. The Gamma pass‐rate was only 59% for the central target and 40% for the peripheral target when no prediction filter was applied. This means that tracking drastically improves the target dose and that a prediction filter improves the dosimetric distribution of tracking during lung SBRT.

We added a 1 mm/min baseline drift to the periodic motion because baseline drifts are often present in respiratory motion.^28^ The additional baseline drift further decreased the GTV coverage ($D_{98\%}$) to 72% for a central target when we did not apply tracking. However, when we applied tracking with a prediction filter, the delivered dose and the target coverage were in excellent agreement with the reference delivery, suggesting that baseline drift did not affect the tracking performance. Small residual dose differences remain between tracking and the reference case because the cross‐correlation method used to identify the target location slightly underestimates the target motion.

A less complex alternative for MLC tracking is tumor trailing.^21^ During trailing, the beam aperture is continuously adjusted according to the target’s last time‐averaged position.^21^ The main advantage of trailing is that it is not affected by system latency or by the low imaging frequency; it therefore does not require a lookahead prediction.^21^ From our dosimetric results, we see that trailing reduces the influence of baseline drift and gives an identical GTV coverage as a midP delivery without baseline drift. From the gamma analysis, we see that trailing during periodic+drift motion provided a dose distribution that is almost in full agreement with a midP dose distribution during periodic motion. This suggests that trailing effectively mitigates the impact of baseline drift for midP deliveries. Thus, trailing can be used to improve conventional midP deliveries in real‐time.

Both tracking and trailing benefit the treatment precision of lung SBRT with smaller treatment margins compared to ITV deliveries, and with real‐time treatment control. Tracking gives the most optimal dosimetric results, but it is associated with a higher degree of technological complexity and the need for a motion predictor. As a minimum, the implementation of trailing is recommended for MRI‐guided lung SBRT to reduce the influence of baseline motion and to enhance the accuracy of midP treatments. The higher treatment precision of both strategies potentially allows to reduce the number of treatment fractions in the future while maintaining tumor control. The dosimetric precision we obtained during MLC tracking shows the potential to reduce the conventional ITV margins used to treat lung tumors.^11^ Smaller treatment margins extend the applicability of lung SBRT because of the risk of critical organ damage.^9^, ^13^, ^14^ This is especially important to increase the applicability for central lung tumors.^9^, ^13^, ^14^

Although our results show the feasibility of both tracking and trailing for lung SBRT, further work is needed before these can be used clinically. In particular, the periodic motion we used for the dosimetry experiments was somewhat simplistic, resulting in very accurate predictions. However, based on our *in silico* predictor results, we do not expect substantial dosimetric degradation for real respiratory traces, as our predictor showed only small residual position errors on real respiratory traces. Furthermore, the large leaf speed (6 cm/s) would also allow to track faster breathing.^10^, ^19^ In this study, we only used one‐dimensional (1D) SI‐directed tumor motion, while lung tumors also, more subtly, move in left‐right and anterior–posterior direction.^19^, ^28^ Additional movements resulting from tumor deformations^32^, ^33^ were also neglected. Tracking tumor motion also in these directions could allow for even smaller PTV margins. In our current study, we neglected these movements due to the limitation of our phantom that can only perform translational motion in SI direction.

Tracking motion in multiple directions, requires a multidimensional predictor. A linear regression predictor can be trivially extended to process multidimensional data by performing independent 1D predictions along each coordinate. Alternatively, a multidimensional training and prediction filter can be applied, in which the size of the multidimensional space can be limited by establishing a common input vector for all three coordinates, as described by Krauss et al. (2011) and Ruan and Keall (2010).^19^, ^30^ Although the latter gives a more accurate prediction,^19^ it might also induce more latency because the calculations are more complex, which could require longer lookahead lengths.

Another limitation of our study is the uniform spherical target we used. Contrasting to our target, real tumors exist in a wide variety of shapes and sizes. Furthermore, they generally have a fuzzy outline on the cine images, which makes it more difficult to identify the tumor during real‐time tracking and trailing. However, previous studies already showed the feasibility of different auto‐contouring algorithms for lung tumors on cine images.^34^ This indicates that MRI‐guided tracking and trailing would also be possible with cine images of real tumors.

## 5 CONCLUSION

We provided a first experimental demonstration of the technical feasibility of MRI‐guided MLC tracking and trailing for central and peripheral lung SBRT. Tracking maximizes the sparing of healthy tissue, while trailing is highly effective in mitigating baseline motion. A linear regression prediction filter, tailored for low‐frequency MRI guidance, mitigates system latency during tracking and substantially reduces dosimetric errors. Furthermore, trailing can narrow the dosimetric gap between MLC tracking and midP deliveries by effectively tracking the baseline motion.
